# Impact of the 2010 Pakistan Floods on Rural and Urban Populations at Six Months

**DOI:** 10.1371/4fdfb212d2432

**Published:** 2012-08-22

**Authors:** Thomas D. Kirsch, Christina Wadhwani, Lauren Sauer, Shannon Doocy, Christina Catlett

## Abstract

The 2010 Pakistan flood affected 20 million people. The impact of the event and recovery is measured at 6 months. Methods: Cross-sectional cluster survey of 1769 households conducted six months post-flood in 29 most-affected districts. The outcome measures were physical damage, flood-related death and illness and changes in income, access to electricity, clean water and sanitation facilities. Results: Households were headed by males, large and poor. The flood destroyed 54.8% of homes and caused 86.8% households to move, with 46.9% living in an IDP camp. Lack of electricity increased from 18.8% to 32.9% (p = 0.000), lack of toilet facilities from 29.0% to 40.4% (p=0.000). Access to protected water remained unchanged (96.8%); however, the sources changed (p=0.000). 88.0% reported loss of income (90.0% rural, 75.0% urban, p=0.000) with rural households loosing significantly more and less likely to recovered. Immediate deaths and injuries were uncommon but 77.0% reported flood-related illnesses. Significant differences were noted between urban and rural as well as gender and education of the head of houshold. Discussion: After 6 months, much of the population had not recovered their prior standard of living or access to services. Rural households were more commonly impacted and slower to recover. Targeting relief to high-risk populations including rural, female-headed and those with lower education is needed. Citation: Kirsch TD, Wadhwani C, Sauer L, Doocy S, Catlett C. Impact of the 2010 Pakistan Floods on Rural and Urban Populations at Six Months. PLOS Currents Disasters. 2012 Aug 22. doi: 10.1371/4fdfb212d2432.

## Introduction

Natural disasters interfere with economy and destroy infrastructure, resulting in a disruption of livelihoods, normal services and health care. Floods can be particularly disruptive, leading to widespread collapse of infrastructure. They are the leading cause of deaths from natural disaster worldwide, with 6.8 million deaths in the 20^th^ century. Asia is the most flood-affected region, accounting for nearly 50% of flood-related fatalities in the last 25 years.^1^ ^2^ Floods and their impact are likely to increase in the future due to urbanization and land use changes, high concentrations of poor and marginalized populations, and a lack of regulations and preparedness efforts.^3^

The 2010 Pakistan floods directly affected an estimated 14-20 million people, and killed over 1,700. Nearly 1.1 million homes were damaged or destroyed, and at least 436 health care facilities were destroyed.^4^ The flooding lasted almost six months in some areas and caused $9.7 billion in damage^5^ in forty-six of the country’s 135 districts. The impact on the rural economy, including agriculture crops, livestock, animal sheds, personal seed stocks, fertilizers, agricultural machinery, fisheries and forestry, was unprecedented.^6^ Infrastructure losses were widespread including 2.9 million damaged households, of which 1.9 were severely affected or completely destroyed, and 80% of food reserves lost.^7^

The general impact of floods has been previously described, but there is little information on the medium-term impact and recovery from flood events. The purpose of this study is to characterize the impact of the 2010 floods on the Pakistani people after six months of humanitarian interventions and the variables that affect recovery. This study explores how predictors such as education, income, family size, and rural versus urban location factor in to flood impact. Furthermore, change in income level and disruption of services (e.g., protected water sources, electricity, and toilet facilities) among the affected population was examined in order to better understand how this influenced post-flood living and household conditions in urban and rural settings.

## Methods

A cross-sectional cluster survey of heads of households affected by the 2010 floods in Pakistan was conducted in January 2011. The Government of Pakistan and the World Bank identified four affected provinces (Balochistan, Khyber Pakhtunkhwa, Punjab, Sindh) with 29 districts designated as “severely” flood-affected.( The survey was focused on these priority districts. There were 80 clusters of 20 households each, with an additional oversample of 10 clusters in camp-based populations. Clusters were chosen based on the proportion of the affected population within provinces and again at the district level. Randomly generated GIS coordinates were then chosen as starting points for each cluster within the most severely-affected tehsils (counties) or union councils (sub-counties).^8^ Three selected clusters were found to be inaccessible due to security concerns or snow-blocked roads and so were re-randomized within the same affected district. The ten camp-based clusters were added to account for the difficulty of randomly selecting these small geographic areas. These ten clusters were distributed on a population proportional basis from an official list of current camps and the size of their registered populations.

A household was defined as a group of people that live in the same living quarters and share meals, regardless of biological relation. The first household in each cluster was identified as the nearest to the GIS point. In rural areas the next nearest house was chosen until all 20 households were surveyed. In urban and camp settings with dense populations, each 5^th^ house was sampled. Households were eligible for the survey if they had been affected by the flood (economic, health or physical damage), if an adult member (>18 years) was present, and if they agreed to participate. If a household was not eligible or not inhabited at the time of the survey, the next closest household was approached.

The sample size was based on the objectives of characterizing the impact of the floods on rural and urban households’ social, economic and health status. The nearly 1800 households allowed for a detection of a ≥6.25% difference in prevalence rates (based on the most conservative prevalence of 50%) after adjusting for a design effect of 1.5 for the cluster sample design. Changes to income level and access to services such as electricity, protected water sources, and toilet facilities were the primary outcome variables of interest.

Data analyses were conducted on STATA version 11 software (STATACorp LP, College Station, Texas). Descriptive statistics and summary measures for each group were calculated, and comparisons were drawn using standard tests (chi square) for statistical significance. The Johns Hopkins School of Public Health Institutional Review Board and the Pakistan Ministry of Health approved the survey. Voluntary informed consent was sought from all survey participants.

## Results

***Pre-Flood Living Conditions***

A total of 1,769 households were included, representing 14,506 people with 87.0% living in a rural setting prior to the flooding. The demographics of the households in our survey closely matched national Pakistani statistics.^9^ The average household size was 8.1 (8.2 for urban, 7.6 rural, p=0.001). Half (50.6%) of household members were under 18 years, while 5.0% were over 60 years. The head of household (HOH) was a male in 96.2% of homes, with an average HOH age of 46.4 years (range 18-96). Household members were more likely to have completed at least primary school education than the HOH (54.1% vs. 34.6%, p=0.00). Urban households had significantly higher education at all levels than rural (p=0.00). Households’ monthly income averaged less than 10,000 Rupees (about $112 USD). Urban populations were more likely to earn less than 5,000 Rupees per month (31.0% vs. 21.8%, p= 0.00).

Mud brick was the most common material for the walls (79.4% overall; 56.2% urban vs. 82.9% rural, p=0.00) and floors (83.7%; 68.1% urban vs. 85.1% rural, p=0.00). While 40.6% of roofs were also made of mud or mud brick (19.7% urban vs. 42.2% rural, p=0.00), cement, wood, and other materials were also used. Prior to the floods, electricity was available to 81.2% of households (88.6% urban vs. 80.1% rural, p=0.001), primarily from power lines. 71.0% of households had access to some type of toilet facility (67.4% urban vs. 71.5% rural, p=0.00). Almost all households (91.8%) had access to a protected water source (87.7% urban vs. 92.5% rural, p=0.00).

***Impact of the Flooding***

Physical damage from the floods was widespread, with 54.8% of households reporting damage to their homes. Over half (54.8%) of these were damaged beyond repair, 28.8% had significant but reparable damage, 10.9% minor but livable damage, and 5.6% with minimal damage. The average household size did not change after the floods in either the rural (7.6 to 7.5, p=0.08) or urban setting (8.2 to 8.0, p=0.14), with 80.4% of households having fewer than 10 members, 18.6% of household with 11-20 members, and 1.1% of household with over 21 members (p=0.57). The floods also caused 86.8% of households (76.9% urban vs. 88.3% rural, p=0.00) to leave their homes for 2 or more weeks. At some point during the six months since the flood, 46.9% had lived in an internally displaced person (IDP) camp (44.0% urban vs. 47.3% rural, p=0.41). Most households (64.5%) stayed in only one place during the 6 months, but 34.5% moved at least once, 21.1% twice, 9.9% 3 times, and 4.5% moved to 4 or more locations. Migration from the home district to a different geographic location was less common (12.6% urban vs. 18.4% rural, p=0.00). At the time of the survey, 51.9% of rural households had returned to their home, compared to 73.9% of urban households (p=0.00).

Table 1 assesses infrastructure by comparing access to utilities for rural and urban household pre- and post-flood. At the time of the survey, the number of households with no access to electricity had increased from 18.8% to 32.9% (p = 0.00). Lack of electricity doubled in both urban and rural areas. The percent of households who did not have access to any toilet facilities increased from 29.0% to 40.4% (p=0.000), but the increase was greater for rural households. The access to protected water after the floods remained unchanged (96.8% before vs. 96.7% after, p=0.00); however, the water source changed significantly in both settings (p=0.00).

**Table 1: Utility Access, Before and Six Months after Floods in Urban and Rural, Pakistan 2010:** 

	Urban					Rural				
	Before		After		p-val	Before		After		p-val
	n	%	n	%		n	%	n	%	
ELECTRICITY	229		228		0.00	1526		1515		0.00
Powelines	175	76%	158	69%		1202	79%	979	65%	
Generator	21	9%	2	1%		10	0.7%	6	0.4%	
Other	7	3%	10	4%		10	0.7%	15	1.0%	
None	26	11%	58	25%		304	20%	515	40%	
	n	%	n	%	p-val	n	%	n	%	p-val
TOILET TYPE	227		222		0.00	1505		1485		
Private	145	64%	109	49%		819	54%	481	32%	
Public/Shared	8	4%	21	10%		257	17%	407	27%	
None	74	33%	92	41%		429	29%	597	40%	
	n	%	n	%	p-val	n	%	n	%	p-val
WATER SOURCE	227		226		0.00	1520		1527		
Indoor	44	19%	37	16%		187	12%	158	10%	0.00
Tanker Truck	6	3%	14	6%		31	2%	236	16%	
Borehole	142	63%	141	62%		1190	78%	1020	67%	
Unprotected Well	2	1%	3	1%		10	1%	26	2%	
River	26	12%	31	14%		96	6%	87	6%	

The flood adversely affected the income of 88.0% of households (90.0% rural, 75.0% urban, p=0.00). The average reported household monthly income dropped from less than 10,000 Rupees per month to less than 5,000 Rupees (about $56 USD). Rural households were more likely to report a monthly salary less than 5,000 Rupees than urban (75.2% vs. 73.1%, p=0.00). Of those households that reported a loss of income, 55.5% stated that their livelihoods or income generation had not returned back to normal after six months and that recovery was expected to be more than an additional year (57.1% rural, urban 42.5, p=0.004). 24.8% of households did not expect to ever recover from income losses.

While injuries and deaths directly related to the floods were rare, health effects were common. Only 0.5% of households reported a flood-related household member injury, and only 0.5% reported a flood-related death. Deaths were significantly more likely in urban households (p=0.02). During the six months post-flood, however, 77.0% of households (71.1% urban vs. 76.5% rural, p=0.02) reported having a member with a health problem, 76.8% of these thought by survey participants to be related to the floods. Urban households reported more flood-related health problems than rural (80.1% vs. 76.5%, p=0.01).

Table 2 assesses variables that predict recovery of services at 6 months. The age of the HOH did not change access to electricity, clean water or toilet facilities. The education level of the HOH did have an impact of access: households headed by a person with primary school education or greater were more likely to have had a return to pre-flood access to electricity and sanitation services, but not clean water. An increased likelihood of access to toilet facilities was also predicted by the gender of the HOH and for households with less than 15 members.

**Table 2: Access to Utilities at Six Months, Controlling for Household Variables, Pakistan 2010:** 

	Electricity			Water			Toilets		
	OR	CI	p-value	OR	CI	p-value	OR	CI	p-value
Household Head Education (Ref=none)	2.3	1.8-2.8	0.00	1.4	0.6-3.0	0.44	1.8	1.5-2.2	0.00
Household Head Gender (Ref=Male)	1.2	0.7-2.0	0.53	0.9	0.1-1.0	0.90	2.1	1.2-3.7	0.01
Household Head Age (Ref >50 years)	1.0	1.0-1.0	0.14	1.0	1.0-1.0	0.53	1.0	1.0-1.0	0.68
Household Size (Ref < 15)	1.2	0.9-1.6	0.28	0.9	0.4-2.3	0.83	1.4	1.0-1.8	0.03

## Discussion

The 2010 Pakistan floods affected as many as 20 million people. Flood waters receded in Balochistan and KPK within days, and after several weeks in Punjab, but months in some areas of Sindh. At the time of this survey there were still four districts of Sindh and one in Punjab with flooded union councils. There was widespread infrastructure damage with resultant loss of housing and services such as electricity, water and sanitation. The economic impact of the floods was also widespread, and our survey demonstrates that even after 6 months much of the population have made limited progress towards their prior standard of living and access to services. Previous reports have looked at specific health-related effects of the 2010 Pakistan floods including infectious diseases, malnutrition^12^, malaria^13^ , cholera and other water born diseases^14^ ; however, to our knowledge none have explored the overall household impact of the floods, the variance and magnitude of the impact between urban and rural settings, or the recovery from the impact at six-months.

The flood caused relatively few direct deaths (1/10,000 affected) and injuries considering, but indirect and long-term health problems were widespread, with 77% of households reporting flood-related injuries or illnesses. These findings are similar to 2005 post-earthquake health needs in Pakistan when 68% of elderly people in rural areas experienced an overall worsening of health^15^ , but our percentage is higher than the injuries and illnesses reported by 31% of households after floods in Nepal in 2009.^16^

The extensive damage to homes, economy and infrastructure identified in our study is consistent with reports on the impact of floods in Nepal in 2008^19^ . However, the widespread damage in Pakistan did not lead to as much distant migration; while nearly 90% of households were forced to leave their homes, and a third moved at least twice, less than 20% moved away from their original geographic region. Only half of the affected households lived in an organized camp at any time since the flood, which would suggest that alternative sites such as other family members’ homes may have been used. However, we found that the average size of both urban and rural households changed minimally at 6 months, contrary to prior reports, where floods and other disasters have been shown to cause a change in household size.^17^

There has been limited research on the differential effects of disasters in rural vs. urban populations.^18^ ^20^ Our study demonstrates significantly worse impact and a slower recovery for rural settings after the flood, including greater impact on their income, sanitation and electricity supply and less frequent economic recovery at six months. Rural households were also more likely to have moved to an entirely different geographic area and less likely to have been able to return to their original home after six months. Rural areas are more difficult to target in a disaster response because of the dispersed nature of the population, but in Pakistan rural households accounted for almost 90% of the total affected. Rural families need increased resources and targeted programs by national and international humanitarian and disaster response agencies.

Economic and infrastructure recovery had been limited at the time of the survey. The education level of a head of household was the most predictive variable for a return to pre-flood access to electricity and sanitation services. This was not significant for clean water, most likely due to the almost universal return of water supplies after the floods (92%). Access to toilet facilities (private or public) was improved by having a male HOH, an HOH with at least a primary school education, and a smaller family. Rural families were less likely to be headed by a person with any formal education, which could contribute to their slower recovery.

## Limitations

There are a number of study limitations. Difficulties in selecting small geographical areas required an oversampling of the IDPs, but accurate populations and proportions could not be ascertained. This may have led to results favoring camp-based populations. This study was conducted six months after the2010 floods. Respondents may have suffered from recall bias related to acute post-disaster experiences, affecting the accuracy of survey responses. Finally, survivor bias may have also been present in the research study. Only houses with surviving adult members were sampled and interviewed. So undercounting would occur if households completely moved out of the area because they would not have been selected.

## Conclusions

Our study identifies the ongoing impact of the 2010 floods on the Pakistani people at 6 months post-disaster and demonstrates the disproportionate and longer-term impact of the flooding on the rural population. It also reaffirms that the less-educated are more likely to suffer the impacts of a disaster. The population’s recovery to pre-flood development has been very slow, with limited improvement at six months. Important lessons learned from this survey are the need to target more vulnerable households, especially those with less education, for additional relief resources. More importantly there is a need to direct relief efforts towards the rural population. This is a more difficult strategy and will require more coordination nationally and locally. Further studies are suggested regarding the link increased illness and rate of recovery in an affected population and the access to clean water and sanitation services.
